# Ethyl 2-(4-chloro­phenyl)-3-(2,4-di­fluoro­phenoxy)acrylate

**DOI:** 10.1107/S1600536808037318

**Published:** 2008-11-20

**Authors:** Hai-Bin Gong, Jie Wang, Ying Liu, Lei Wang

**Affiliations:** a Xuzhou Central Hospital, Xuzhou Cardiovascular Disease Institute, Xuzhou 221009, People’s Republic of China

## Abstract

In the mol­ecule of the title compound, C_17_H_13_ClF_2_O_3_, the dihedral angles formed by the aromatic rings of the chloro­benzene and difluoro­benzene groups with the plane of the acrylate unit are 48.85 (12) and 9.07 (14)°, respectively. In the crystal structure, mol­ecules are linked by weak inter­molecular C—H⋯O hydrogen-bond inter­actions, forming chains along the *c* axis.

## Related literature

For the synthesis and crystal structures of related compounds, see: Li, Xue *et al.* (2008 ▶); Li, Wang & Jian (2008 ▶); Lin & Jian (2008 ▶); Liu *et al.* (2008 ▶). For bond-length data, see: Allen *et al.* (1987 ▶).
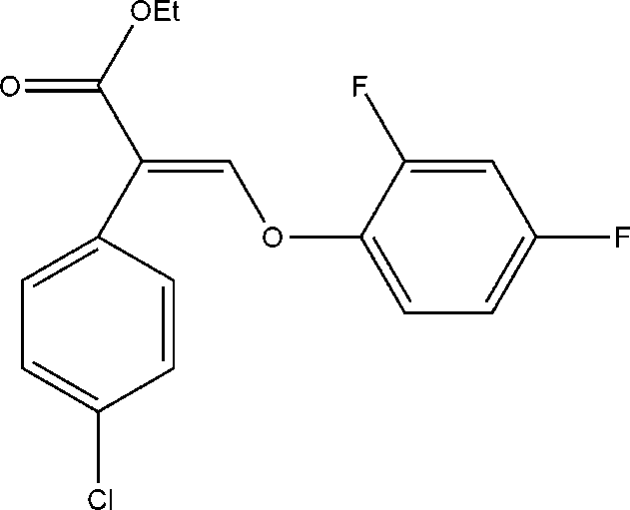


## Experimental

### 

#### Crystal data

C_17_H_13_ClF_2_O_3_
*M* *_r_* = 338.72
Monoclinic, 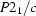

*a* = 16.275 (3) Å
*b* = 7.503 (2) Å
*c* = 13.812 (3) Å
β = 111.11 (3)°
*V* = 1573.4 (7) Å^3^
*Z* = 4
Mo *K*α radiation
μ = 0.28 mm^−1^
*T* = 298 (2) K
0.30 × 0.10 × 0.10 mm

#### Data collection

Bruker SMART 1000 CCD area-detector diffractometer
Absorption correction: multi-scan (*SADABS*; Bruker, 2001 ▶) *T*_min_ = 0.922, *T*_max_ = 0.973
2956 measured reflections
2823 independent reflections
1566 reflections with *I* > 2σ(*I*)
*R*_int_ = 0.027

#### Refinement

*R*[*F* ^2^ > 2σ(*F* ^2^)] = 0.065
*wR*(*F* ^2^) = 0.189
*S* = 1.02
2823 reflections
210 parameters
H-atom parameters constrained
Δρ_max_ = 0.38 e Å^−3^
Δρ_min_ = −0.34 e Å^−3^

### 

Data collection: *SMART* (Bruker, 2007 ▶); cell refinement: *SAINT* (Bruker, 2007 ▶); data reduction: *SAINT*; program(s) used to solve structure: *SHELXTL* (Sheldrick, 2008 ▶); program(s) used to refine structure: *SHELXTL*; molecular graphics: *SHELXTL*; software used to prepare material for publication: *SHELXTL*.

## Supplementary Material

Crystal structure: contains datablocks global, I. DOI: 10.1107/S1600536808037318/rz2266sup1.cif

Structure factors: contains datablocks I. DOI: 10.1107/S1600536808037318/rz2266Isup2.hkl

Additional supplementary materials:  crystallographic information; 3D view; checkCIF report

## Figures and Tables

**Table 1:** Hydrogen-bond geometry (Å, °)

*D*—H⋯*A*	*D*—H	H⋯*A*	*D*⋯*A*	*D*—H⋯*A*
C6—H6⋯O1^i^	0.93	2.51	3.321 (4)	146

Symmetry code: (i) 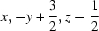.

**Fig. 1.**
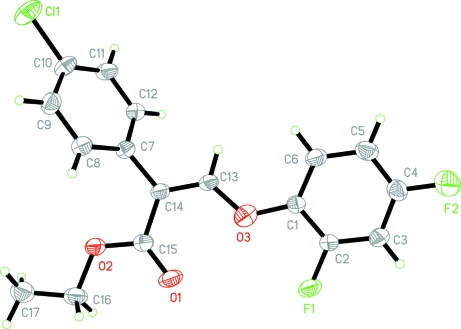
The molecular structure of the title compound, showing 30% probability displacement ellipsoids and the atom-numbering scheme.

## supplementary crystallographic information

### Comment

Recently, the synthesis and structure of a number of etheric compounds have been widely investigated (Li, Xue *et al.*, 2008; Li, Wang & Jian, 2008; Lin & Jian, 2008; Liu *et al.*, 2008). We report herein the crystal structure of the new title compound.

In the molecule of the title compound (Fig. 1), the dihedral angles between the aromatic rings of the chlorobenzene and difluorobenzene groups with the plane of the acrylate unit are 48.85 (12) and 9.07 (14)° respectively. All the bond lengths (Allen *et al.*, 1987) and angles are not unusual. In the crystal structure, molecules are linked by weak intermolecular C—H···O hydrogen interactions forming chains along the *c* axis (Table 1).

### Experimental

An equimolar solution of ethyl 3-bromo-2-(4-chlorophenyl)acrylate and 2,4-difluorophenol in chloroform was left to react overnight at room temperature. Block-shaped crystals suitable for X-ray analysis were obtained by slow evaporation of the solvent in air for five days.

### Refinement

H atoms were included in the riding model approximation with C–H = 0.93–0.97 Å and with *U*_iso_(H) = 1.2 *U*_eq_(C) or 1.5 *U*_eq_(C) for methyl H atoms.

### Crystal data

**Table:**

C_17_H_13_ClF_2_O_3_	*F*_000_ = 696
*M**_r_* = 338.72	*D*_x_ = 1.430 Mg m^−^^3^
Monoclinic, *P*2_1_/*c*	Mo *K*α radiation λ = 0.71073 Å
Hall symbol: -P 2ybc	Cell parameters from 1002 reflections
*a* = 16.275 (3) Å	θ = 2.4–24.5º
*b* = 7.503 (2) Å	µ = 0.28 mm^−^^1^
*c* = 13.812 (3) Å	*T* = 298 (2) K
β = 111.11 (3)º	Block, colorless
*V* = 1573.4 (7) Å^3^	0.30 × 0.10 × 0.10 mm
*Z* = 4	

### Data collection

**Table:**

Bruker SMART 1000 CCD area-detector diffractometer	2823 independent reflections
Radiation source: fine-focus sealed tube	1566 reflections with *I* > 2σ(*I*)
Monochromator: graphite	*R*_int_ = 0.027
*T* = 298(2) K	θ_max_ = 25.2º
ω scans	θ_min_ = 1.3º
Absorption correction: multi-scan(SADABS; Bruker, 2001)	*h* = −19→18
*T*_min_ = 0.922, *T*_max_ = 0.973	*k* = −9→10
2956 measured reflections	*l* = −16→16

### Refinement

**Table:**

Refinement on *F*^2^	Hydrogen site location: inferred from neighbouring sites
Least-squares matrix: full	H-atom parameters constrained
*R*[*F*^2^ > 2σ(*F*^2^)] = 0.065	*w* = 1/[σ^2^(*F*_o_^2^) + (0.0904*P*)^2^] where *P* = (*F*_o_^2^ + 2*F*_c_^2^)/3
*wR*(*F*^2^) = 0.189	(Δ/σ)_max_ = 0.001
*S* = 1.02	Δρ_max_ = 0.38 e Å^−^^3^
2823 reflections	Δρ_min_ = −0.34 e Å^−^^3^
210 parameters	Extinction correction: SHELXTL (Sheldrick, 2008), Fc^*^=kFc[1+0.001xFc^2^λ^3^/sin(2θ)]^-1/4^
Primary atom site location: structure-invariant direct methods	Extinction coefficient: 0.018 (3)
Secondary atom site location: difference Fourier map	

### Special details

**Table:**

Geometry. All e.s.d.'s (except the e.s.d. in the dihedral angle between two l.s. planes) are estimated using the full covariance matrix. The cell e.s.d.'s are taken into account individually in the estimation of e.s.d.'s in distances, angles and torsion angles; correlations between e.s.d.'s in cell parameters are only used when they are defined by crystal symmetry. An approximate (isotropic) treatment of cell e.s.d.'s is used for estimating e.s.d.'s involving l.s. planes.
Refinement. Refinement of *F*^2^ against ALL reflections. The weighted *R*-factor *wR* and goodness of fit *S* are based on *F*^2^, conventional *R*-factors *R* are based on *F*, with *F* set to zero for negative *F*^2^. The threshold expression of *F*^2^ > σ(*F*^2^) is used only for calculating *R*-factors(gt) *etc*. and is not relevant to the choice of reflections for refinement. *R*-factors based on *F*^2^ are statistically about twice as large as those based on *F*, and *R*- factors based on ALL data will be even larger.

### Fractional atomic coordinates and isotropic or equivalent isotropic displacement parameters (Å^2^)

**Table:**

	*x*	*y*	*z*	*U*_iso_*/*U*_eq_	
C1	1.0187 (2)	0.7796 (5)	0.9699 (3)	0.0450 (9)	
C2	1.1022 (2)	0.7724 (5)	1.0453 (3)	0.0473 (9)	
C3	1.1764 (2)	0.8337 (6)	1.0319 (3)	0.0581 (11)	
H3	1.2315	0.8266	1.0844	0.070*	
C4	1.1654 (3)	0.9066 (6)	0.9368 (3)	0.0597 (11)	
C5	1.0851 (3)	0.9178 (6)	0.8598 (3)	0.0621 (12)	
H5	1.0797	0.9691	0.7964	0.074*	
C6	1.0121 (3)	0.8535 (6)	0.8755 (3)	0.0561 (11)	
H6	0.9574	0.8596	0.8222	0.067*	
C7	0.7054 (2)	0.6469 (5)	0.8670 (3)	0.0423 (9)	
C8	0.6325 (2)	0.7234 (5)	0.8808 (3)	0.0540 (10)	
H8	0.6376	0.7663	0.9459	0.065*	
C9	0.5527 (2)	0.7366 (6)	0.7996 (3)	0.0571 (11)	
H9	0.5045	0.7876	0.8100	0.069*	
C10	0.5451 (2)	0.6742 (5)	0.7036 (3)	0.0543 (11)	
C11	0.6147 (3)	0.5952 (6)	0.6881 (3)	0.0572 (11)	
H11	0.6086	0.5513	0.6229	0.069*	
C12	0.6947 (2)	0.5804 (5)	0.7696 (3)	0.0482 (10)	
H12	0.7418	0.5252	0.7588	0.058*	
C13	0.8635 (2)	0.7022 (5)	0.9293 (3)	0.0465 (9)	
H13	0.8526	0.7373	0.8612	0.056*	
C14	0.7936 (2)	0.6482 (5)	0.9511 (3)	0.0426 (9)	
C15	0.8065 (2)	0.5946 (5)	1.0579 (3)	0.0483 (10)	
C16	0.7414 (3)	0.4744 (6)	1.1715 (3)	0.0627 (12)	
H16A	0.7580	0.5754	1.2185	0.075*	
H16B	0.7858	0.3824	1.1975	0.075*	
C17	0.6537 (3)	0.4055 (7)	1.1643 (3)	0.0716 (13)	
H17A	0.6093	0.4922	1.1303	0.107*	
H17B	0.6538	0.3831	1.2327	0.107*	
H17C	0.6415	0.2968	1.1250	0.107*	
Cl1	0.44478 (7)	0.69754 (19)	0.60039 (9)	0.0891 (5)	
F1	1.10978 (13)	0.7012 (3)	1.13855 (15)	0.0663 (7)	
F2	1.23761 (16)	0.9699 (4)	0.9209 (2)	0.0858 (9)	
O3	0.94846 (18)	0.7113 (4)	0.9954 (2)	0.0723 (9)	
O1	0.87704 (17)	0.6033 (4)	1.13049 (18)	0.0666 (9)	
O2	0.73412 (16)	0.5282 (4)	1.06775 (17)	0.0525 (7)	

### Atomic displacement parameters (Å^2^)

**Table:**

	*U*^11^	*U*^22^	*U*^33^	*U*^12^	*U*^13^	*U*^23^
C1	0.043 (2)	0.048 (2)	0.043 (2)	−0.0032 (18)	0.0138 (16)	−0.0068 (18)
C2	0.047 (2)	0.057 (2)	0.0348 (19)	0.0045 (19)	0.0106 (16)	−0.0028 (18)
C3	0.042 (2)	0.078 (3)	0.049 (2)	−0.007 (2)	0.0090 (17)	−0.010 (2)
C4	0.053 (2)	0.070 (3)	0.062 (3)	−0.008 (2)	0.028 (2)	−0.011 (2)
C5	0.071 (3)	0.074 (3)	0.044 (2)	−0.001 (2)	0.024 (2)	0.001 (2)
C6	0.049 (2)	0.073 (3)	0.040 (2)	−0.005 (2)	0.0084 (17)	−0.002 (2)
C7	0.042 (2)	0.043 (2)	0.039 (2)	−0.0041 (17)	0.0112 (15)	0.0051 (16)
C8	0.048 (2)	0.062 (3)	0.046 (2)	0.002 (2)	0.0106 (17)	−0.004 (2)
C9	0.044 (2)	0.063 (3)	0.060 (3)	0.007 (2)	0.0137 (19)	0.004 (2)
C10	0.044 (2)	0.056 (3)	0.048 (2)	−0.003 (2)	−0.0007 (17)	0.0099 (19)
C11	0.059 (2)	0.067 (3)	0.038 (2)	−0.006 (2)	0.0081 (18)	−0.001 (2)
C12	0.048 (2)	0.054 (2)	0.041 (2)	−0.0005 (19)	0.0140 (16)	−0.0013 (18)
C13	0.045 (2)	0.055 (2)	0.0329 (18)	0.0044 (19)	0.0056 (16)	0.0002 (17)
C14	0.042 (2)	0.047 (2)	0.0327 (18)	0.0010 (17)	0.0072 (15)	−0.0008 (16)
C15	0.049 (2)	0.052 (2)	0.039 (2)	−0.0023 (19)	0.0113 (18)	−0.0016 (18)
C16	0.069 (3)	0.076 (3)	0.038 (2)	−0.006 (2)	0.0140 (19)	0.002 (2)
C17	0.075 (3)	0.084 (3)	0.057 (3)	−0.013 (3)	0.025 (2)	−0.001 (2)
Cl1	0.0572 (7)	0.1132 (11)	0.0671 (8)	0.0017 (7)	−0.0137 (5)	0.0169 (7)
F1	0.0541 (13)	0.0976 (19)	0.0380 (12)	−0.0022 (13)	0.0054 (10)	0.0107 (12)
F2	0.0689 (16)	0.119 (2)	0.0845 (18)	−0.0260 (16)	0.0462 (14)	−0.0119 (17)
O3	0.0592 (18)	0.086 (2)	0.0644 (19)	−0.0014 (17)	0.0134 (15)	−0.0021 (17)
O1	0.0509 (16)	0.099 (2)	0.0377 (15)	−0.0153 (16)	0.0018 (12)	0.0063 (15)
O2	0.0487 (15)	0.0678 (18)	0.0382 (13)	−0.0050 (14)	0.0123 (11)	0.0020 (13)

### Geometric parameters (Å, °)

**Table:**

C1—C6	1.384 (5)	C10—C11	1.363 (5)
C1—C2	1.385 (5)	C10—Cl1	1.747 (4)
C1—O3	1.409 (4)	C11—C12	1.385 (5)
C2—F1	1.358 (4)	C11—H11	0.9300
C2—C3	1.365 (5)	C12—H12	0.9300
C3—C4	1.373 (6)	C13—C14	1.340 (5)
C3—H3	0.9300	C13—O3	1.356 (4)
C4—F2	1.357 (4)	C13—H13	0.9300
C4—C5	1.359 (5)	C14—C15	1.469 (5)
C5—C6	1.370 (5)	C15—O1	1.224 (4)
C5—H5	0.9300	C15—O2	1.331 (4)
C6—H6	0.9300	C16—O2	1.452 (4)
C7—C12	1.386 (5)	C16—C17	1.486 (5)
C7—C8	1.392 (5)	C16—H16A	0.9700
C7—C14	1.486 (4)	C16—H16B	0.9700
C8—C9	1.380 (5)	C17—H17A	0.9600
C8—H8	0.9300	C17—H17B	0.9600
C9—C10	1.368 (5)	C17—H17C	0.9600
C9—H9	0.9300		
			
C6—C1—C2	116.5 (3)	C10—C11—C12	119.9 (4)
C6—C1—O3	125.9 (3)	C10—C11—H11	120.1
C2—C1—O3	117.6 (3)	C12—C11—H11	120.1
F1—C2—C3	118.6 (3)	C11—C12—C7	120.8 (4)
F1—C2—C1	117.2 (3)	C11—C12—H12	119.6
C3—C2—C1	124.1 (4)	C7—C12—H12	119.6
C2—C3—C4	116.6 (4)	C14—C13—O3	127.3 (3)
C2—C3—H3	121.7	C14—C13—H13	116.3
C4—C3—H3	121.7	O3—C13—H13	116.3
F2—C4—C5	119.7 (4)	C13—C14—C15	118.8 (3)
F2—C4—C3	118.2 (4)	C13—C14—C7	119.1 (3)
C5—C4—C3	122.1 (4)	C15—C14—C7	122.1 (3)
C4—C5—C6	119.8 (4)	O1—C15—O2	122.7 (3)
C4—C5—H5	120.1	O1—C15—C14	124.1 (4)
C6—C5—H5	120.1	O2—C15—C14	113.2 (3)
C5—C6—C1	120.9 (4)	O2—C16—C17	107.1 (3)
C5—C6—H6	119.6	O2—C16—H16A	110.3
C1—C6—H6	119.6	C17—C16—H16A	110.3
C12—C7—C8	117.8 (3)	O2—C16—H16B	110.3
C12—C7—C14	120.6 (3)	C17—C16—H16B	110.3
C8—C7—C14	121.4 (3)	H16A—C16—H16B	108.5
C9—C8—C7	121.1 (4)	C16—C17—H17A	109.5
C9—C8—H8	119.4	C16—C17—H17B	109.5
C7—C8—H8	119.4	H17A—C17—H17B	109.5
C10—C9—C8	119.6 (4)	C16—C17—H17C	109.5
C10—C9—H9	120.2	H17A—C17—H17C	109.5
C8—C9—H9	120.2	H17B—C17—H17C	109.5
C11—C10—C9	120.7 (3)	C13—O3—C1	124.9 (3)
C11—C10—Cl1	120.0 (3)	C15—O2—C16	116.4 (3)
C9—C10—Cl1	119.2 (3)		
			
C6—C1—C2—F1	179.6 (3)	C10—C11—C12—C7	−0.7 (6)
O3—C1—C2—F1	−0.6 (5)	C8—C7—C12—C11	2.1 (6)
C6—C1—C2—C3	0.4 (6)	C14—C7—C12—C11	−173.4 (4)
O3—C1—C2—C3	−179.8 (4)	O3—C13—C14—C15	1.2 (6)
F1—C2—C3—C4	−179.2 (3)	O3—C13—C14—C7	−179.6 (3)
C1—C2—C3—C4	0.0 (6)	C12—C7—C14—C13	44.5 (5)
C2—C3—C4—F2	179.6 (4)	C8—C7—C14—C13	−130.9 (4)
C2—C3—C4—C5	0.1 (7)	C12—C7—C14—C15	−136.4 (4)
F2—C4—C5—C6	179.8 (4)	C8—C7—C14—C15	48.3 (5)
C3—C4—C5—C6	−0.7 (7)	C13—C14—C15—O1	3.5 (6)
C4—C5—C6—C1	1.1 (7)	C7—C14—C15—O1	−175.7 (4)
C2—C1—C6—C5	−1.0 (6)	C13—C14—C15—O2	−174.1 (3)
O3—C1—C6—C5	179.3 (4)	C7—C14—C15—O2	6.8 (5)
C12—C7—C8—C9	−1.6 (6)	C14—C13—O3—C1	−175.8 (4)
C14—C7—C8—C9	173.9 (4)	C6—C1—O3—C13	2.4 (6)
C7—C8—C9—C10	−0.2 (6)	C2—C1—O3—C13	−177.4 (4)
C8—C9—C10—C11	1.6 (6)	O1—C15—O2—C16	3.3 (6)
C8—C9—C10—Cl1	−178.1 (3)	C14—C15—O2—C16	−179.1 (3)
C9—C10—C11—C12	−1.2 (6)	C17—C16—O2—C15	179.9 (3)
Cl1—C10—C11—C12	178.6 (3)		

### Hydrogen-bond geometry (Å, °)

**Table:**

*D*—H···*A*	*D*—H	H···*A*	*D*···*A*	*D*—H···*A*
C6—H6···O1^i^	0.93	2.51	3.321 (4)	146

Symmetry codes: (i) *x*, −*y*+3/2, *z*−1/2.
